# Suppressive Effects of Bee Venom Acupuncture on Paclitaxel-Induced Neuropathic Pain in Rats: Mediation by Spinal α_2_-Adrenergic Receptor

**DOI:** 10.3390/toxins9110351

**Published:** 2017-10-31

**Authors:** Jiho Choi, Changhoon Jeon, Ji Hwan Lee, Jo Ung Jang, Fu Shi Quan, Kyungjin Lee, Woojin Kim, Sun Kwang Kim

**Affiliations:** 1 Department of Physiology, College of Korean Medicine, Kyung Hee University, 26 Kyungheedae-ro, Dongdamoon-gu, Seoul 02447, Korea; cyanical@hotmail.com (J.C.); cjmystars44@gmail.com (C.J.); 2 Department of Science in Korean Medicine, Graduate School, Kyung Hee University, 26 Kyungheedae-ro, Dongdamoon-gu, Seoul 02447, Korea; mibdna@khu.ac.kr; 3 Department of East-West Medicine, Graduate School, Kyung Hee University, 26 Kyungheedae-ro, Dongdamoon-gu, Seoul 02447, Korea; powerfox032@naver.com; 4 Department of Medical Zoology, School of Medicine, Kyung Hee University, 26 Kyungheedae-ro, Dongdamoon-gu, Seoul 02447, Korea; fsquan@khu.ac.kr; 5 Department of Herbology, College of Korean Medicine, Kyung Hee University, 26 Kyungheedae-ro, Dongdamoon-gu, Seoul 02447, Korea; niceday@khu.ac.kr

**Keywords:** bee venom acupuncture, chemotherapy-induced neuropathic pain, paclitaxel

## Abstract

Paclitaxel, a chemotherapy drug for solid tumors, induces peripheral painful neuropathy. Bee venom acupuncture (BVA) has been reported to have potent analgesic effects, which are known to be mediated by activation of spinal α-adrenergic receptor. Here, we investigated the effect of BVA on mechanical hyperalgesia and spinal neuronal hyperexcitation induced by paclitaxel. The role of spinal α-adrenergic receptor subtypes in the analgesic effect of BVA was also observed. Administration of paclitaxel (total 8 mg/kg, intraperitoneal) on four alternate days (days 0, 2, 4, and 6) induced significant mechanical hyperalgesic signs, measured using a von Frey filament. BVA (1 mg/kg, ST36) relieved this mechanical hyperalgesia for at least two hours, and suppressed the hyperexcitation in spinal wide dynamic range neurons evoked by press or pinch stimulation. Both melittin (0.5 mg/kg, ST36) and phospholipase A2 (0.12 mg/kg, ST36) were shown to play an important part in this analgesic effect of the BVA, as they significantly attenuated the pain. Intrathecal pretreatment with the α_2_-adrenergic receptor antagonist (idazoxan, 50 µg), but not α_1_-adrenergic receptor antagonist (prazosin, 30 µg), blocked the analgesic effect of BVA. These results suggest that BVA has potent suppressive effects against paclitaxel-induced neuropathic pain, which were mediated by spinal α_2_-adrenergic receptor.

## 1. Introduction

Paclitaxel is an important chemotherapeutic agent from the bark of *Taxus brevifolia* [1], which is widely used to treat various tumors [2,3,4]. However, despite its role against the tumors, its usage is often limited, due to the painful peripheral neuropathy occurring after its administration [5]. Symptoms commonly reported are sensory neuropathies, which are paresthesia, loss of tendon reflexes, numbness and pain in the upper and lower extremities. Although these neuropathies decrease patients’ quality of life (QoL), there is still no optimal treatment method or drug to alleviate these neuropathies [5,6]. Thus, an effort to explore novel treatments is needed.

Bee venom acupuncture (BVA), a treatment method that injects diluted bee venom into acupoints, is widely used in traditional Korean medicine against various diseases, such as adhesive capsulitis [7], idiopathic Parkinson’s disease [8], knee osteoarthritis [9], and musculoskeletal pain diseases [10]. Especially, BVA has been reported to have potent analgesic effect in studies conducted using various animal models of pain [11,12,13,14,15], and two case series also reported that BVA treatment may help to reduce the chemotherapy-induced peripheral neuropathy (CIPN), including paclitaxel-induced neuropathy [16,17]. Recently, our laboratory has demonstrated that BVA treatment could significantly alleviate mechanical and cold allodynia in a rat model of oxaliplatin-induced neuropathic pain [12,14,18,19]. Moreover, although the precise mechanism of BVA analgesic effect is unknown, we have also demonstrated that this analgesic effect was mediated by the descending noradrenergic pain modulation pathway via the activation of spinal α-adrenergic receptor, which was consistent with other previously conducted studies [11,12,13,14,20].

Thus, the aims of this study were, firstly, to examine whether the BVA has suppressive effects against paclitaxel-induced mechanical hyperalgesia and neuronal hyperexcitation in the spinal cord, and secondly, to observe the role of BVA components, such as melittin and phospholipase A2 (PLA2) in their analgesic effect, and finally, to investigate which α-adrenergic receptor subtypes mediate the analgesic effect of BVA in the spinal cord.

## 2. Results

### 2.1. Development and Maintanance of Paclitaxel-Induced Mechanical Hyperalgesia

**Figure 1:**
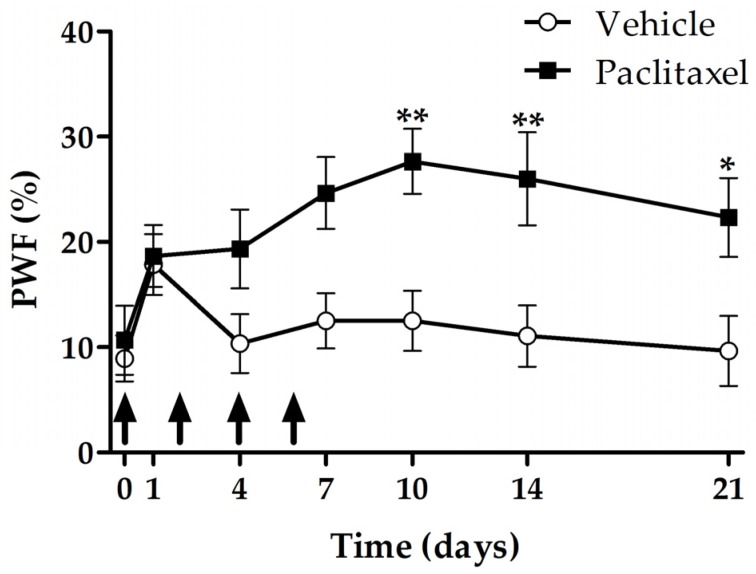
Time course of paclitaxel-induced mechanical hyperalgesia. Rats were divided into two groups; paclitaxel (*n* = 7), vehicle (*n* = 7). Paclitaxel (2.0 mg/kg per injection) or vehicle was injected to rats four times (arrows; days 0, 2, 4 and 6). Significant differences between two groups were observed from the day 10 to day 21. Data are presented as mean ± SEM (* *p* < 0.05, ** *p* < 0.01; two-way ANOVA followed by Bonferroni’s multiple comparison test).

In order to see the time-elapsed change of paclitaxel-induced mechanical hyperalgesia, we evaluated the withdrawal responses of hind paws to mechanical stimulation using a von Frey filament with 15 g bending force. In the paclitaxel group, significant increase in paw withdrawal frequency (PWF) was shown from 10 to 21 days after the first injection (*p* < 0.01, day 10 and 14; *p* < 0.05, day 21) (Figure 1). Therefore, we performed the following experiments on day 10 through to 21.

### 2.2. Effects of BVA on Paclitaxel-Induced Mechanical Hyperalgesia

**Figure 2:**
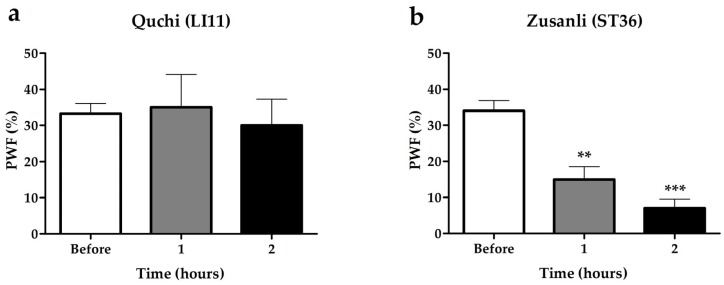
Effects of bee venom acupuncture (BVA) at different acupoints on paclitaxel-induced mechanical hyperalgesia. BVA (1.0 mg/kg) was used at (**a**) LI11 (*n* = 6) or (**b**) ST36 (*n* = 5) acupoints. In ST36 group, the paw withdrawal frequency (PWF) decreased significantly one or two hours after BVA, whereas no significant differences are shown in LI11 group. Data are presented as mean ± SEM (** *p* < 0.01, *** *p* < 0.001; repeated measures one-way ANOVA followed by Dunnett’s post hoc test).

**Figure 3:**
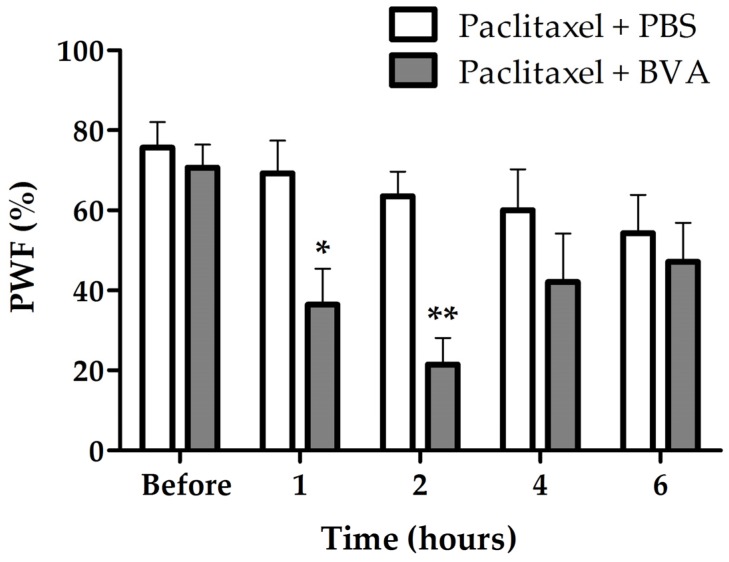
Time course of the analgesic effect of BVA on paclitaxel-induced mechanical hyperalgesia. Rats were dispensed arbitrarily into two groups; paclitaxel + BVA (*n* = 7), paclitaxel + phosphate buffered saline (PBS) (*n* = 7). BVA (1.0 mg/kg) and PBS were treated at ST36. Significant reduction of PWF was observed from one to two hours after BVA. Data are presented as mean ± SEM (* *p* < 0.05, ** *p* < 0.01; two-way ANOVA followed by Bonferroni’s multiple comparison test).

Since the BVA treatment at Zusanli (ST36) acupoint, but not at Quchi (L11), showed significant anti-hyperalgesic effect (Figure 2), BVA was used at ST36 in the following experiments. Figure 3 shows the analgesic effects of BVA on paclitaxel-induced mechanical hyperalgesia with the time course. BVA treated group (paclitaxel + BVA) showed significant reduction in PWF compared to control group (paclitaxel + PBS (phosphate buffered saline)) at one and two hours after BVA (47% reduction, *p* < 0.05 and 66% reduction, *p* < 0.01, respectively). No significant difference between the two groups was shown from four hours after BVA. These results indicate that the treatment of BVA has a potent analgesic effect on paclitaxel-induced neuropathic pain, lasting at least two hours.

### 2.3. Effects of BVA on Paclitaxel-Induced Hyperexcitation in the Spinal Wide Dynamic Range (WDR) Neurons

**Figure 4:**
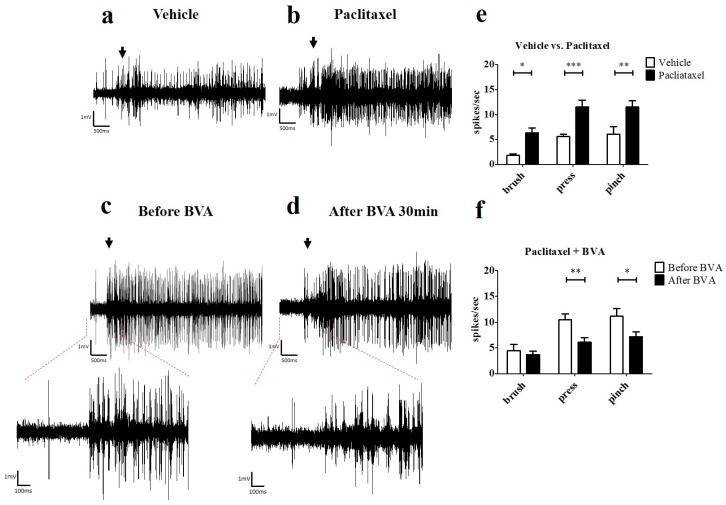
Paclitaxel-induced hyperexcitation in wide dynamic range (WDR) neurons and inhibition of paclitaxel-induced hyperexcitation by BVA treatment. (**a**–**d**) Representative extracellular recording raw traces of WDR neuron’s responses to pressing with hard stick (arrows, during 5 s) in vehicle group (**a**), paclitaxel group (**b**), and BVA (1 mg/kg) treated group (**c**,**d**). Before BVA treatment (**c**) and 30 min after BVA treatment (**d**). (**e**,**f**) The spike response of WDR neurons to mechanical stimulation (brush, press, and pinch). Data are presented as mean ± SEM (* *p* < 0.05, ** *p* < 0.01, *** *p* < 0.001; two-way ANOVA followed by Bonferroni’s multiple comparison test).

In order to see whether paclitaxel induces hyperexcitation in WDR neurons and BVA treatment reduces paclitaxel-induced hyperexcitation in WDR neurons, we conducted extracellular recording in vivo (Figure 4a–d). The number of spike responses of WDR neurons to mechanical stimulation (brush, press, and pinch) was significantly increased in paclitaxel group (*p* < 0.05; brush, *p* < 0.001; press and pinch, vs. vehicle, Figure 4e). In the BVA treatment group (1 mg/kg, ST36), significant reduction of paclitaxel-induced hyperexcitation in WDR neurons was observed (*p* < 0.01; press, *p* < 0.001; pinch, vs. before BVA, Figure 4f).

### 2.4. Effect of BVA, Melittin, or PLA2 on Paclitaxel-Induced Mechanical Hyperalgesia

**Figure 5:**
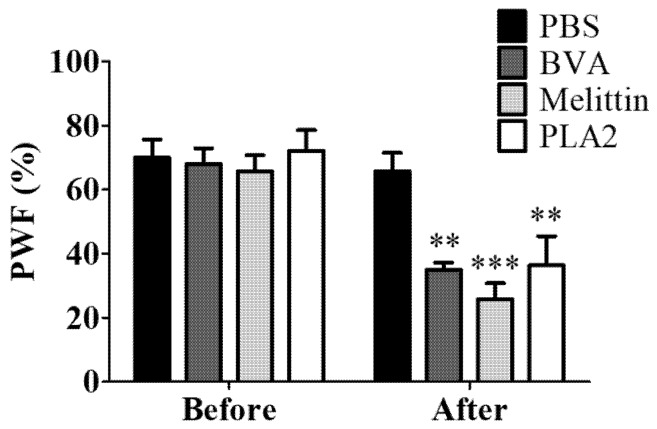
The analgesic effect of BVA, melittin, or PLA2 on paclitaxel-induced mechanical hyperalgesia. Rats showing signs of mechanical allodynia were dispensed arbitrarily into four groups; PBS (*n* = 7), BVA (1 mg/kg, *n* = 5), melittin (0.5 mg/kg, *n* = 6), and PLA2 (0.12 mg/kg, *n* = 7). All drugs were injected at ST36. PBS was used as control. Behavioral tests were conducted one hour after the drug administrations. Data are presented as mean ± SEM (** *p* < 0.01, *** *p* < 0.001; two-way ANOVA followed by Bonferroni’s multiple comparison test).

To observe the role of different BV components in the analgesic effect of the BVA, BVA (1 mg/kg), melittin (0.5 mg/kg), or PLA2 (0.12 mg/kg) were injected at ST36. The two major protein components of the honey bee are melittin and PLA2, which occupies 50 and 12% of its dry weight, respectively [21]. Behavioral assessments were conducted one hour after the injection of BVA, melittin, or PLA2, as BVA showed its strongest analgesic effect one hour after the injection (Figure 3). This result showed that melittin had a stronger analgesic effect against paclitaxel-induced mechanical hyperalgesia than BVA or PLA2 (Figure 5).

### 2.5. Effects of Intrathecal α-Adrenergic Receptor Subtype Antagonists on BVA- or Melittin-Induced Anti-Hyperalgesia

**Figure 6:**
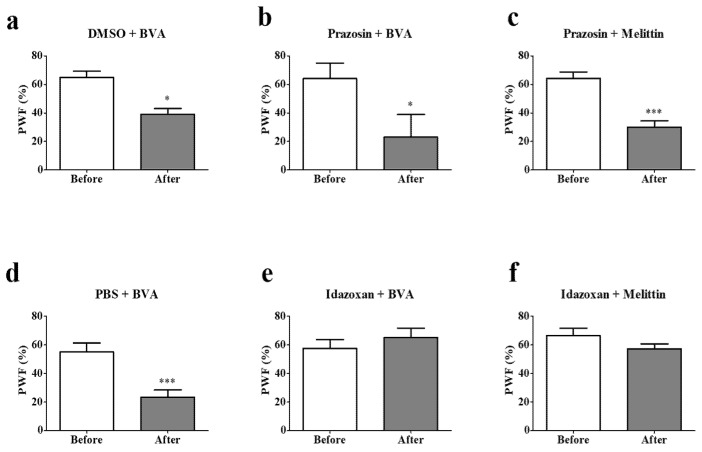
Effects of intrathecal adrenergic antagonists on BVA- or melittin-induced analgesic action. Rats were divided into six groups; (**a**) DMSO + BVA (*n* = 5), (**b**) prazosin + BVA (*n* = 5), (**c**) prazosin + melittin (*n* = 7), (**d**) PBS + BVA (*n* = 6), (**e**) idazoxan + BVA (*n* = 6), (**f**) idazoxan + melittin (*n* = 7). Data are presented as mean ± SEM (* *p* < 0.05, *** *p* < 0.001; paired *t*-test).

To investigate which α-adrenergic receptor subtypes mediate BVA- or melittin-induced anti-hyperalgesic action, prazosin (α_1_-adrenergic receptor antagonist, 30 μg, i.t.) or idazoxan (α_2_-adrenergic receptor antagonist, 50 μg, i.t.) was administered 20 min before treatments. Prazosin and dimethyl sulfoxide (DMSO) showed significant decrease in PWF after BVA or melittin treatments (Figure 6a–c). This demonstrate that neither BVA nor melittin acted on spinal α_1_-adrenergic receptor to reduce the hyperalgesia evoked by paclitaxel. In contrast, idazoxan, but not PBS (*p* < 0.001), blocked the BVA- or melittin-induced anti-hyperalgesic effect (Figure 6d–f). These results altogether, indicate that the spinal α_2_-adrenergic receptor, but not the α_1_-adrenergic receptor, mediates BVA- or melittin-induced analgesia.

## 3. Discussion

Multiple injection of paclitaxel can occur peripheral neuropathy, which can limit its usage and decreases patients’ QoL. Although the treatments such as gabapentin, pregabalin, and morphine have been used to alleviate the neuropathic pain, these treatments have, themselves, various side effects, such as nausea, vomiting, somnolence, dizziness, suicidal thought, and drug dependence [22,23,24,25]. Therefore, an effort to search for effective treatment options is critically needed. In traditional Korean medicine, BVA has been used to treat musculoskeletal pain and arthritis from the past [10,26]. In addition, these days, BVA has also been founded to be effective in treating patients with CIPN [16,17]. Thus, in this study, we experimented to find out whether BVA can alleviate the paclitaxel-induced neuropathy and to clarify the mechanism that lies behind it.

Our data showed that BVA treatment at ST36, not LI11, had a significant analgesic effect. It should be noted that ST36 acupoint is closer to the hind paw, where mechanical test was performed, than LI11 acupoint. It is consistent with the previous study in which BVA had more potent analgesic effect when treated closer to the tested area [14]. Then, we examined the time course of the analgesic effect of the BVA at ST36. The result showed that the analgesic effect was significant until two hours after BVA treatment. Our previous study also showed that the analgesic effect of BVA was effective until two hours after BVA treatment in oxaliplatin-induced cold allodynia [14]. Considering that moderate concentration of morphine without side effects was no longer effective in oxaliplatin-induced cold allodynia at two hours after administration [12,27], this result would be clinically significant.

The spinal wide dynamic range (WDR) neuron receives non-nociceptive and nociceptive inputs via A- and C-fibers, and descending pain modulatory systems synapse at the WDR neuron [28]. Therefore, the spinal WDR neuron is suitable for assessing the degree of pain. In addition, the hyperexcitation of spinal WDR neuron was observed previously in a rat model of paclitaxel-induced hyperalgesia [29]. In our study, electrophysiological data confirmed that hyperexcitation of WDR neurons is induced by paclitaxel. We further demonstrated that BVA treatment could significantly inhibit this paclitaxel-induced hyperexcitation in the spinal WDR cells.

In subsequent experiments, we administered BVA, melittin, or PLA2 at ST 36, to observe the role of different BV components in the analgesic effect of BVA against paclitaxel-induced mechanical hyperalgesia. Melittin is a major component of the BV, occupying 50% of its total dry weight. PLA2 occupies 12%. Our results showed that 0.5 mg/kg of melittin was more powerful than 1 mg/kg of BVA or 0.12 mg/kg of PLA2. In our previous study, we showed that intraperitoneal injection of PLA2 could significantly decrease the cold and mechanical allodynia induced by single oxaliplatin injection in mice [30]. Moreover, although not on chemotherapy induced pain model, other lab has reported that melittin injected at ST36 had a powerful analgesic effect against complete Freund’s adjuvant-induced rheumatoid arthritis, showing a similar effect to BVA [31]. In this study, the analgesic effect of BVA or melittin was blocked by spinal α_2_-adrenergic receptor antagonist (idazoxan), showing that BVA and melittin act on similar spinal adrenergic receptors to inhibit mechanical hyperalgesia induced by paclitaxel.

EA (electro-acupuncture) is a modified acupuncture which utilizes electrical current to treat pain. BVA is another form of acupuncture which uses chemical compounds; bee venom. The two different forms of acupuncture have similarities and differences. One of the similarities is that the endogenous analgesic systems are involved in both of their analgesic mechanisms, and the difference is that the analgesic effects of EA are mainly mediated by the opioidergic system [32], whereas those of the BVA are mostly mediated by the noradrenergic system [33]. However, despite this difference, EA and BVA were both reported to be effective in different types of allodynia assessed using thermal [34] and chemical [12] stimulations. These results show that other inhibitory systems, such as serotonergic, GABA, and/or cholinergic systems, may also play an important role, along with opioidergic and adrenergic system, in the action of EA and BVA. Furthermore, interaction of periaqueductal gray (PAG) and locus coeruleus (LC) in the brain should also play an important part in their analgesic effect, as both the EA and BVA were reported to activate PAG [35] and LC [36], which are important opioid and noradrenaline producing site in the CNS, respectively.

BVA induced analgesia was shown to be mediated by spinal α_2_-adrenergic receptor [11,12,13,14,15], and it increased c-Fos expression in LC and A5 cell group (A5) [37,38]. Moreover, BVA reduced c-Fos expression in the spinal dorsal horn of rats with formalin or acetic acid-induced pain [13,39]. Considering that both the LC and A5 are part of the descending noradrenergic pathway [40], it is suggested that BVA suppresses conduction of afferent nociceptive signals in the spinal dorsal horn affecting descending noradrenergic pathway. Our data are consistent with previous studies showing that spinal α_2_-adrenergic receptor mediates BVA-induced analgesia. Furthermore, the pain attenuating effect of melittin was also blocked by spinal α_2_-adrenergic receptor antagonist (idazoxan) showing that melittin, the richest component of the BV, also acts on spinal α_2_-adrenergic receptor to inhibit mechanical hyperalgesia induced by paclitaxel.

Drug combination is widely used to treat dreadful diseases, such as AIDS and cancer. The main aim of drug combination is to reduce dose and toxicity, and to delay the induction of drug resistance. Our previous study showed the combined effect of BVA and morphine on oxaliplatin-induced neuropathic pain [12]. BVA treated with morphine showed prolonged analgesic effects compared to the BVA or morphine alone. Moreover, another article showed that BVA could enhance the analgesic effect of intrathecal injection of clonidine in chronic constriction injury-induced neuropathic pain model [41]. Because such combined effect on paclitaxel-induced neuropathic pain has yet to be studied, further studies are needed to examine the combined effect of BVA with other drugs, like morphine, clonidine, SSRI, SNRI, gabapentin, and cannabinoid. Furthermore, in the future studies, it will be interesting to investigate the effect of various components of the BVA on paclitaxel-induced neuropathic pain model, as several active components exist in the BV, such as melittin [42] and PLA2 [30], which have been reported to be effective in other pain models.

## 4. Conclusions

In conclusion, BVA (1 mg/kg) at ST36 significantly attenuated mechanical hyperalgesia induced by paclitaxel. The significant analgesic effect lasted two hours, which was long enough compared to the effect of morphine. Suppressive action was verified by conducting extracellular recording in the spinal WDR neurons. Moreover, both melittin (0.5 mg/kg) and PLA2 (0.12 mg/kg), which are major components of the BV, significantly attenuated the paclitaxel-induced mechanical hyperalgesia. This analgesic effect of BVA or melittin was significantly blocked by intrathecal injection of idazoxan, but not by prazosin, demonstrating that the action of spinal α_2_-adrenergic receptor, but not α_1_-adrenergic receptor, is involved in the mechanism of analgesic effect.

## 5. Materials and Methods

### 5.1. Animals

Adult Sprague-Dawley rats (male, 180–210 g, 6 weeks old) (Daehan Biolink, Chungbuk, Korea) were housed in cages with free access to food and water, and were sustained at 23 ± 2 °C room temperature with a 12 hour light/dark cycle. Prior to any experiments, all animals were acclimated in their cages (3–4 rats per cage) for a week. All experiments using animals were ratified by the Institutional Animal Care and Use Committee of Kyung Hee University (KHUASP(SE)-16-153), and were performed on the ground of the guidelines of the International Association for the Study of Pain [43].

### 5.2. Administration of Paclitaxel

Paclitaxel (Wako Pure Chemical Industries, Osaka, Japan) was dissolved in cremophor EL polyethoxylated castor oil (Sigma, St. Louis, MO, USA) and 100% ethanol (Merck KGaA, Marmstadt, Germany) (1:1 solution), and 6 mg/mL stocks were made. Then, stocks were diluted by phosphate buffered saline (PBS) at a concentration of 2 mg/ml and administrated at an amount of 2 mg/kg on four alternate days (days 0, 2, 4, and 6). As control, the same volume of vehicle was intraperitoneally injected. The formula of paclitaxel was slightly modified from previous studies [44,45].

### 5.3. Behavior Tests

Twenty to thirty minutes before the behavior test, animals were adapted to the experimental circumstances. The experimenters were blinded to paclitaxel and any other treatments. The animals were placed on a metal mesh, enclosed within a 20 (d) × 20 (w) × 14 (h) cm clear plastic cage. Mechanical hyperalgesia was assessed using von Frey filament (Stoelting Co., Wood Dale, IL, USA). The measurement method of mechanical hyperalgesia was modified from the previous studies [44,45,46]. On the mid-plantar area of both hind paws, the von Frey filament (bending force of 15 g) was stimulated for 10 times each, with the applications held for 5 s. The percentage of withdrawal responses to the von Frey filament application was calculated, and then expressed as an overall percentage response.

### 5.4. Experimental Schedule

**Figure 7:**
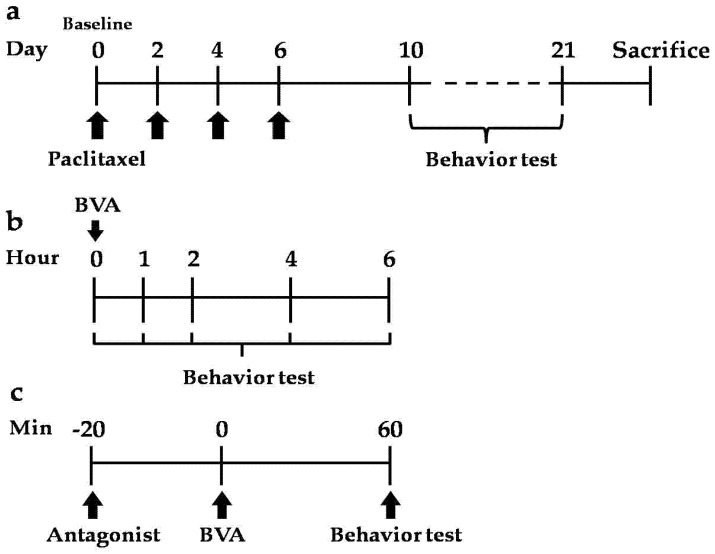
Time schedule of the experiment. (**a**) Paclitaxel was administered four alternate days (0, 2, 4, 6 days, i.p.); (**b**) the time course of BVA effect was conducted at 1, 2, 4, and 6 hours after administration of BVA; (**c**) antagonists were treated 20 min before administration of BVA or melittin, and behavior tests were conducted one hour after administration of BVA or melittin.

The time schedule of this experiment is shown in Figure 7. After baseline mechanical sensitivity was measured at day 0, paclitaxel was injected intraperitoneally on four alternate days (days 0, 2, 4, and 6) (Figure 7a). Behavior tests were performed after paclitaxel administration. The time course of BVA effect was measured at 1, 2, 4, and 6 hours after administration of BVA (Figure 7b). Antagonists were treated 20 min before BVA, and then, behavior tests were conducted 1 hour later (Figure 7c).

### 5.5. BVA, Melittin, or PLA2 Treatment

To verify the optimal acupoint for the BVA treatment, paclitaxel administered rats were divided randomly into two groups; Quchi (LI11) and Zusanli (ST36). LI11 is located at the depression medial to the extensor carpi radialis, at the lateral end of the cubital crease. ST36 is located in the anterior tibial muscle, 5 mm lateral and distal from the anterior tubercle of the tibia [47].

BV was manufactured by Jayeonsaeng TJ (Kyeonggi-Do, Korea), and its quality is strictly controlled by regular HPLC analysis (SNU National Instrumentation Center for Environmental Management, Seoul, Korea; see Supplementary Materials Figure S1). BV (1.0 mg/kg), as reported as an effective concentration without side effects from a previous study [12], dissolved in PBS was respectively injected at right side LI11 or ST36 acupoints subcutaneously, after baseline mechanical sensitivity was measured. The mechanical behavior test was performed following time course schedule (Figure 7b). Melittin (0.5 mg/kg) and PLA2 (0.12 mg/kg) were also injected at ST36. All drugs injected at acupoints were injected subcutaneously.

### 5.6. Extracellular Recording

Extracellular recordings were made from animals 10–21 days following administration of paclitaxel, when rats exhibited significant mechanical hyperalgesia. Extracellular recordings were carried out as previously described [48]. In brief, rats were anesthetized with urethane (Sigma, St. Louis, MO, USA; 1.5 g/kg, i.p.). The spinal cords of animals, which were fixed in a stereotaxic frame, were exposed from T13–L2 and irrigated with oxygenated (95% O_2_–5% CO_2_ gas) Krebs solution (in mM: 117 NaCl, 3.6 KCl, 2.5 CaCl_2_, 1.2 MgCl_2_, 1.2 NaH_2_PO_4_, 11 glucose, and 25 NaHCO_3_) at a flow rate of 10 to 15 mL/min at 38 ± 1 °C. By their responses to brush, pressure, and pinch, WDR cells were classified. Cells were isolated in the L3–L5 segments medial to the dorsal root entry zone up to a depth of 1000 mm. Extracellular single-unit recordings were made with a low-impedance insulated tungsten microelectrode (impedance of 10 MΩ, FHC, Bowdoin, ME, USA).

For mechanical stimuli, brush, press, and pinch stimulation were applied to the lateral and ventral surfaces of the hind paw. Brush stimulus was given by brushing the receptive field five times with a camel brush. Press stimulus was given by pressing the receptive field five seconds using the blunt tip of the camel brush with a diameter of 0.5 cm and a magnitude of about 20 g. Finally, pinch stimulation was given by pinching the skin using toothed forceps (11022-14, Fine Science Tools, Heidelberg, Germany) for five seconds.

### 5.7. Antagonists

To investigate the mechanism of BVA, paclitaxel administered rats were divided randomly into four groups: dimethyl sulfoxide (DMSO; Sigma, St. Louis, MO, USA) + BVA, prazosin + BVA, prazosin + melittin, PBS + BVA, idazoxan + BVA, and idazoxan + melittin. α_1_-Adrenergic receptor antagonist prazosin (Sigma, St. Louis, MO, USA; 30 μg) was dissolved in 20% DMSO. α_2_-Adrenergic receptor antagonist idazoxan (Sigma; 50 μg) was dissolved in PBS. Under isoflurane anesthesia (Hana Pharm. Co., Kyeonggi-Do, Korea), all antagonists were treated intrathecally with a direct lumbar puncture as previously described [12,48].

### 5.8. Statistical Analysis

All the data are presented as mean ± SEM. Statistical analysis and graphic works were performed with Prism 5.0 (GraphPad software, La Jolla, CA, USA, 2008). Paired *t*-test, one-way ANOVA followed by Dunnett’s post hoc test, and two-way ANOVA followed by Bonferroni’s multiple comparison test were used for statistical analysis. In all cases, *p* < 0.05 was considered significant.

## Supplementary Materials

The following are available online at www.mdpi.com/2072-6651/9/11/351/s1, Figure S1: Representative HPLC analysis of melittin and phospholipase A2 (PLA2) in BV.

## References

[1] Wani M.C., Taylor H.L., Wall M.E., Coggon P., McPhail A.T. (1971). Plant antitumor agents. Vi. Isolation and structure of taxol, a novel antileukemic and antitumor agent from taxus brevifolia. J. Am. Chem. Soc..

[2] Sparano J.A. (2000). Taxanes for breast cancer: An evidence-based review of randomized phase ii and phase iii trials. Clin. Breast Cancer.

[3] Goffin J., Lacchetti C., Ellis P.M., Ung Y.C., Evans W.K. (2010). First-line systemic chemotherapy in the treatment of advanced non-small cell lung cancer: A systematic review. J. Thorac. Oncol..

[4] Covens A., Carey M., Bryson P., Verma S., Fung Kee Fung M., Johnston M. (2002). Systematic review of first-line chemotherapy for newly diagnosed postoperative patients with stage ii, iii, or iv epithelial ovarian cancer. Gynecol. Oncol..

[5] Lee J.J., Swain S.M. (2006). Peripheral neuropathy induced by microtubule-stabilizing agents. J. Clin. Oncol..

[6] Argyriou A.A., Koltzenburg M., Polychronopoulos P., Papapetropoulos S., Kalofonos H.P. (2008). Peripheral nerve damage associated with administration of taxanes in patients with cancer. Crit. Rev. Oncol. Hematol..

[7] Koh P.S., Seo B.K., Cho N.S., Park H.S., Park D.S., Baek Y.H. (2013). Clinical effectiveness of bee venom acupuncture and physiotherapy in the treatment of adhesive capsulitis: A randomized controlled trial. J. Shoulder Elbow Surg..

[8] Cho S.Y., Shim S.R., Rhee H.Y., Park H.J., Jung W.S., Moon S.K., Park J.M., Ko C.N., Cho K.H., Park S.U. (2012). Effectiveness of acupuncture and bee venom acupuncture in idiopathic parkinson’s disease. Parkinsonism Relat. Disord..

[9] Kwon Y.-B., Kim J.-H., Yoon J.-H., Lee J.-D., Han H.-J., Mar W.-C., Beitz A.J., Lee J.-H. (2001). The analgesic efficacy of bee venom acupuncture for knee osteoarthritis: A comparative study with needle acupuncture. J. Chin. Med..

[10] Lee M.S., Pittler M.H., Shin B.C., Kong J.C., Ernst E. (2008). Bee venom acupuncture for musculoskeletal pain: A review. J. Pain.

[11] Kim H.W., Kwon Y.B., Han H.J., Yang I.S., Beitz A.J., Lee J.H. (2005). Antinociceptive mechanisms associated with diluted bee venom acupuncture (apipuncture) in the rat formalin test: Involvement of descending adrenergic and serotonergic pathways. Pharmacol. Res..

[12] Kim W., Kim M., Go D., Min B.-I., Na H., Kim S. (2016). Combined effects of bee venom acupuncture and morphine on oxaliplatin-induced neuropathic pain in mice. Toxins.

[13] Kwon Y.-B., Kang M.-S., Han H.-J., Beitz A.J., Lee J.-H. (2001). Visceral antinociception produced by bee venom stimulation of the zhongwan acupuncture point in mice: Role of α_2_ adrenoceptors. Neurosci. Lett..

[14] Lim B.S., Moon H.J., Li D.X., Gil M., Min J.K., Lee G., Bae H., Kim S.K., Min B.I. (2013). Effect of bee venom acupuncture on oxaliplatin-induced cold allodynia in rats. Evid. Based Complement. Altern. Med. eCAM.

[15] Roh D.H., Kwon Y.B., Kim H.W., Ham T.W., Yoon S.Y., Kang S.Y., Han H.J., Lee H.J., Beitz A.J., Lee J.H. (2004). Acupoint stimulation with diluted bee venom (apipuncture) alleviates thermal hyperalgesia in a rodent neuropathic pain model: Involvement of spinal alpha 2-adrenoceptors. J. Pain.

[16] Park J.W., Jeon J.H., Yoon J., Jung T.Y., Kwon K.R., Cho C.K., Lee Y.W., Sagar S., Wong R., Yoo H.S. (2012). Effects of sweet bee venom pharmacopuncture treatment for chemotherapy-induced peripheral neuropathy: A case series. Integr. Cancer Ther..

[17] Yoon J., Jeon J.H., Lee Y.W., Cho C.K., Kwon K.R., Shin J.E., Sagar S., Wong R., Yoo H.S. (2012). Sweet bee venom pharmacopuncture for chemotherapy-induced peripheral neuropathy. J. Acupunct. Meridian Stud..

[18] Lee J.H., Li D.X., Yoon H., Go D., Quan F.S., Min B.I., Kim S.K. (2014). Serotonergic mechanism of the relieving effect of bee venom acupuncture on oxaliplatin-induced neuropathic cold allodynia in rats. BMC Complement. Altern. Med..

[19] Yoon H., Kim M.J., Yoon I., Li D.X., Bae H., Kim S.K. (2015). Nicotinic acetylcholine receptors mediate the suppressive effect of an injection of diluted bee venom into the gv3 acupoint on oxaliplatin-induced neuropathic cold allodynia in rats. Biol. Pharm. Bull..

[20] Baek Y.H., Huh J.E., Lee J.D., Choi D.Y., Park D.S. (2006). Antinociceptive effect and the mechanism of bee venom acupuncture (apipuncture) on inflammatory pain in the rat model of collagen-induced arthritis: Mediation by alpha2-adrenoceptors. Brain Res..

[21] Eze O.B., Nwodo O.F., Ogugua V.N. (2016). Therapeutic effect of honey bee venom. Proteins (enzymes).

[22] Gilron I., Bailey J.M., Tu D., Holden R.R., Weaver D.F., Houlden R.L. (2005). Morphine, gabapentin, or their combination for neuropathic pain. N. Engl. J. Med..

[23] Ormseth M.J., Scholz B.A., Boomershine C.S. (2011). Duloxetine in the management of diabetic peripheral neuropathic pain. Patient Prefer. Adher..

[24] Serpell M.G. (2002). Gabapentin in neuropathic pain syndromes: A randomised, double-blind, placebo-controlled trial. Pain.

[25] Vinik A.I., Casellini C.M. (2013). Guidelines in the management of diabetic nerve pain: Clinical utility of pregabalin. Diabetes Metab. Syndr. Obes. Targets Ther..

[26] Son D.J., Lee J.W., Lee Y.H., Song H.S., Lee C.K., Hong J.T. (2007). Therapeutic application of anti-arthritis, pain-releasing, and anti-cancer effects of bee venom and its constituent compounds. Pharmacol. Ther..

[27] Ling B., Coudore F., Decalonne L., Eschalier A., Authier N. (2008). Comparative antiallodynic activity of morphine, pregabalin and lidocaine in a rat model of neuropathic pain produced by one oxaliplatin injection. Neuropharmacology.

[28] Baron R., Binder A., Wasner G. (2010). Neuropathic pain: Diagnosis, pathophysiological mechanisms, and treatment. Lancet Neurol..

[29] Cata J.P., Weng H.R., Chen J.H., Dougherty P.M. (2006). Altered discharges of spinal wide dynamic range neurons and down-regulation of glutamate transporter expression in rats with paclitaxel-induced hyperalgesia. Neuroscience.

[30] Li D., Lee Y., Kim W., Lee K., Bae H., Kim S.K. (2015). Analgesic effects of bee venom derived phospholipase a2 in a mouse model of oxaliplatin-induced neuropathic pain. Toxins.

[31] Li J., Ke T., He C., Cao W., Wei M., Zhang L., Zhang J.-X., Wang W., Ma J., Wang Z.-R. (2010). The anti-arthritic effects of synthetic melittin on the complete freund’s adjuvant-induced rheumatoid arthritis model in rats. Am. J. Chin. Med..

[32] Zhang R., Lao L., Ren K., Berman B.M. (2014). Mechanisms of acupuncture–electroacupuncture on persistent pain. Anesthesiology.

[33] Chen J., Lariviere W.R. (2010). The nociceptive and anti-nociceptive effects of bee venom injection and therapy: A double-edged sword. Prog. Neurobiol..

[34] Gim G.-T., Lee J.-h., Park E., Sung Y.-H., Kim C.-J., Hwang W.-w., Chu J.-P., Min B.-I. (2011). Electroacupuncture attenuates mechanical and warm allodynia through suppression of spinal glial activation in a rat model of neuropathic pain. Brain Res. Bull..

[35] Liu W.-C., Feldman S.C., Cook D.B., Hung D.-L., Xu T., Kalnin A.J., Komisaruk B.R. (2004). Fmri study of acupuncture-induced periaqueductal gray activity in humans. Neuroreport.

[36] Kwon Y.-b., Kang M.-s., Ahn C.-j., Han H.-j., Ahn B.-c., Lee J.-h. (2000). Effect of high or low frequency electroacupuncture on the cellular actitivy of catecholaminergic neurons in the brain stem. Acupunct. Electro Ther. Res..

[37] Young Bae K., Ho Jae H., Alvin J.B., Jang Hern L. (2004). Bee venom acupoint stimulation increases fos expression in catecholaminergic neurons in the rat brain. Mol. Cells.

[38] Kwon Y.B., Yoon S.Y., Kim H.W., Roh D.H., Kang S.Y., Ryu Y.H., Choi S.M., Han H.J., Lee H.J., Kim K.W. (2006). Substantial role of locus coeruleus-noradrenergic activation and capsaicin-insensitive primary afferent fibers in bee venom’s anti-inflammatory effect. Neurosci. Res..

[39] Kim H.-W., Kwon Y.-B., Ham T.-W., Roh D.-H., Yoon S.-Y., Lee H.-J., Han H.-J., Yang I.-S., Beitz A.J., Lee J.-H. (2003). Acupoint stimulation using bee venom attenuates formalin-induced pain behavior and spinal cord fos expression in rats. J. Vet. Med. Sci..

[40] Jones S.L., Barnes C.D., Pompeiano O. (1991). Chapter 29—descending noradrenergic influences on pain. Progress in Brain Research.

[41] Yoon S.Y., Roh D.H., Kwon Y.B., Kim H.W., Seo H.S., Han H.J., Lee H.J., Beitz A.J., Lee J.H. (2009). Acupoint stimulation with diluted bee venom (apipuncture) potentiates the analgesic effect of intrathecal clonidine in the rodent formalin test and in a neuropathic pain model. J. Pain.

[42] Lin L., Zhu B.-P., Cai L. (2017). Therapeutic effect of melittin on a rat model of chronic prostatitis induced by complete freund’s adjuvant. Biomed. Pharmacother..

[43] Zimmermann M. (1983). Ethical guidelines for investigations of experimental pain in conscious animals. Pain.

[44] Flatters S.J., Bennett G.J. (2004). Ethosuximide reverses paclitaxel- and vincristine-induced painful peripheral neuropathy. Pain.

[45] Polomano R.C., Mannes A.J., Clark U.S., Bennett G.J. (2001). A painful peripheral neuropathy in the rat produced by the chemotherapeutic drug, paclitaxel. Pain.

[46] Flatters S.J.L., Xiao W.-H., Bennett G.J. (2006). Acetyl-l-carnitine prevents and reduces paclitaxel-induced painful peripheral neuropathy. Neurosci. Lett..

[47] Yin C.S., Jeong H.S., Park H.J., Baik Y., Yoon M.H., Choi C.B., Koh H.G. (2008). A proposed transpositional acupoint system in a mouse and rat model. Res. Vet. Sci..

[48] Choi S., Yamada A., Kim W., Kim S.K., Furue H. (2016). Noradrenergic inhibition of spinal hyperexcitation elicited by cutaneous cold stimuli in rats with oxaliplatin-induced allodynia: Electrophysiological and behavioral assessments. J. Physiol. Sci. JPS.

