# Supplementary material for: Suppressive Effects of Bee Venom Acupuncture on Paclitaxel-Induced Neuropathic Pain in Rats: Mediation by Spinal α2-Adrenergic Receptor
Source: Toxins (Basel). 2017 Oct 31;9(11):351. doi: 10.3390/toxins9110351 (PMC5705966; doi:10.3390/toxins9110351)
Supplement: Supplementary file 1 [file toxins-09-00351-s001.pdf]

Supplementary Materials: Suppressive Effects of Bee Venom Acupuncture on Paclitaxel-Induced Neuropathic Pain in Rats: Mediation by Spinal α_2-Adrenergic Receptor

Jiho Choi, Changhoon Jeon, Ji Hwan Lee, Jo Ung Jan, Fu Shi Quan, Kyungjin Lee, Woojin Kim and Sun Kwang Kim

Standard-Melittin

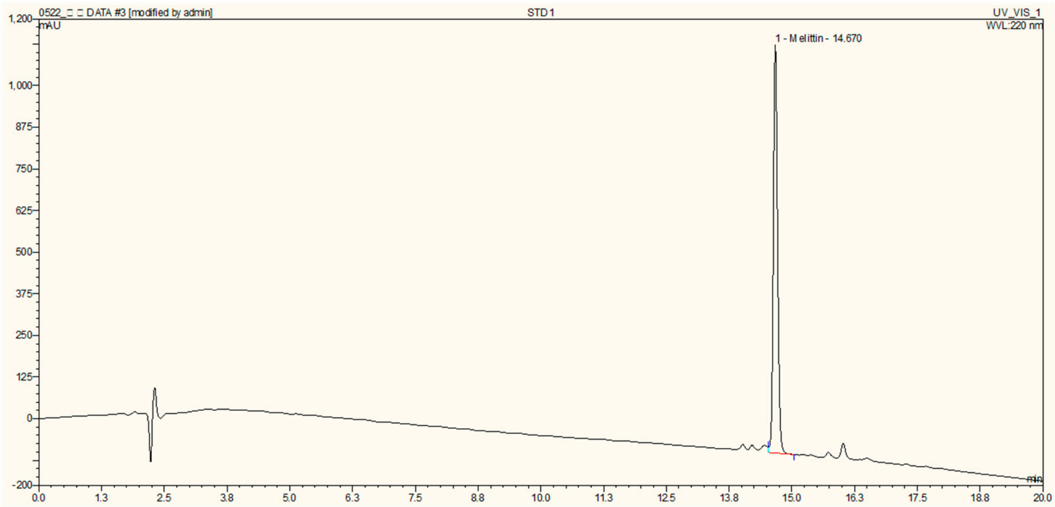

Sample-BV

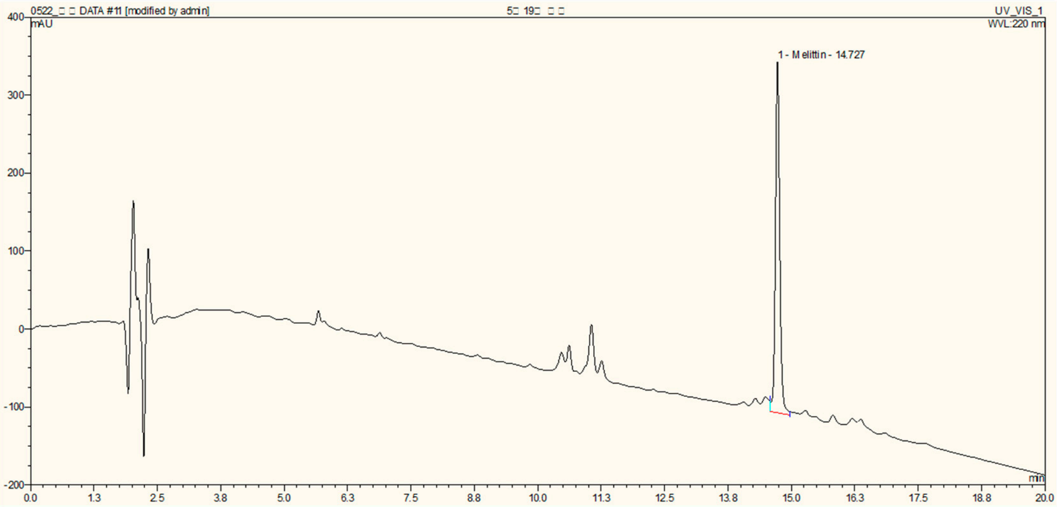

Standard-PLA2

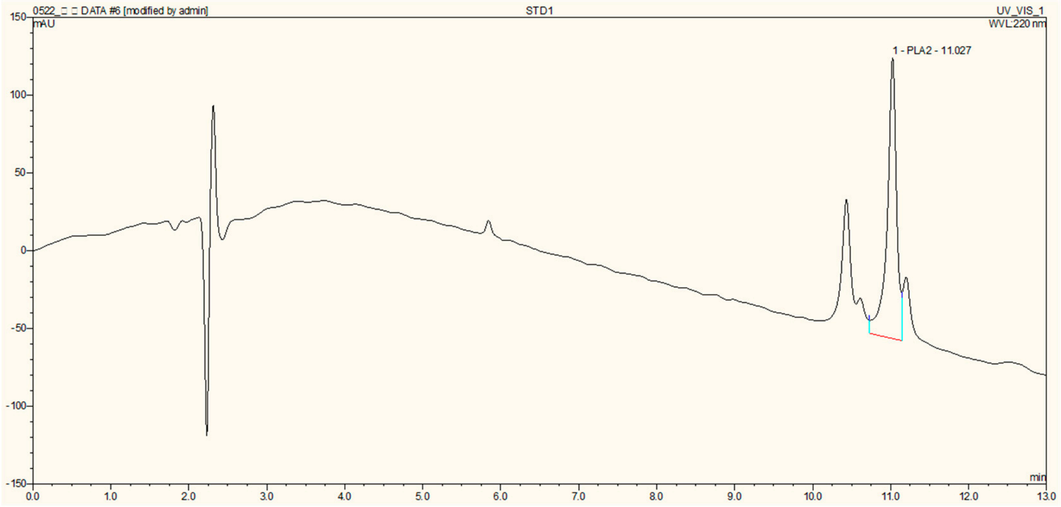

Sample-BV

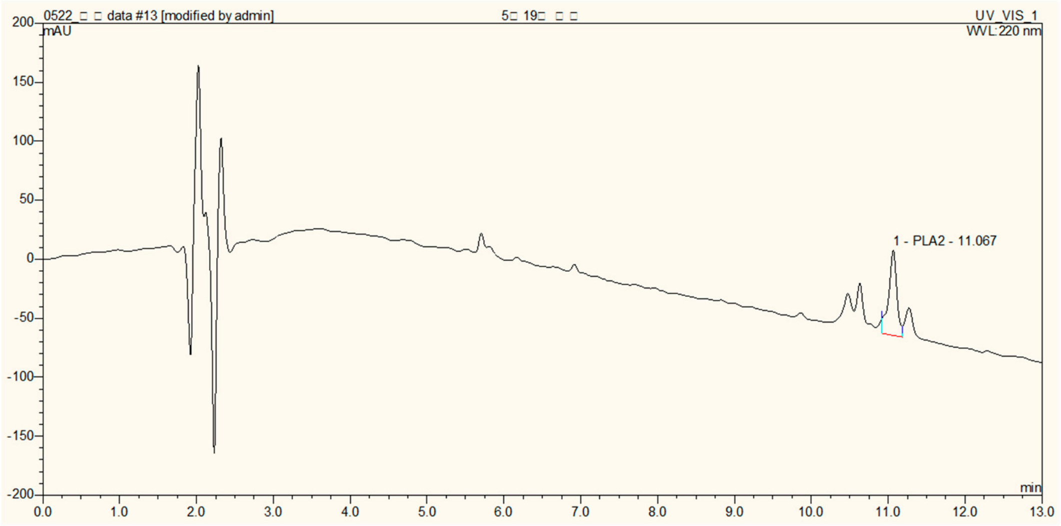

Figure S1. Representative HPLC analysis of Melittin and Phospholipase A2 (PLA2) in BV.

External Calibration Curve

PLA2: $y = 0.0569x + 0.4401$ (r^2=0.999959)

Melittin: y= $0.1272x + 0.1407$ (r^2=0.999973)

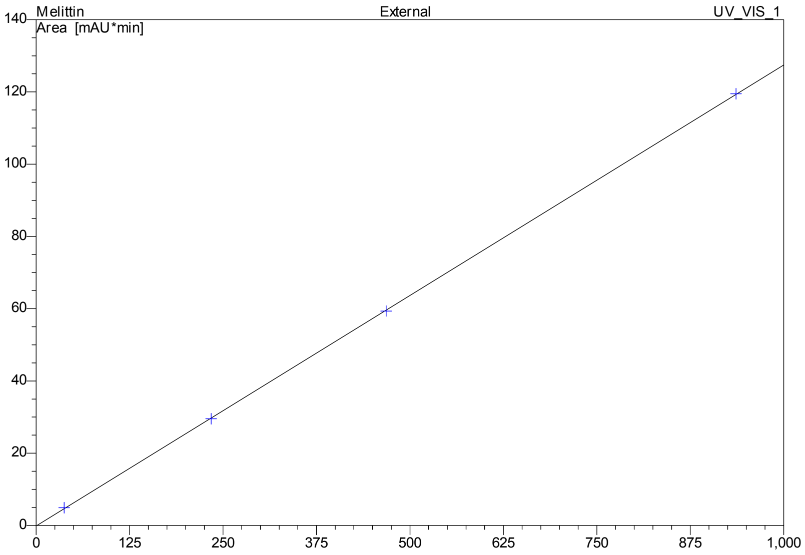

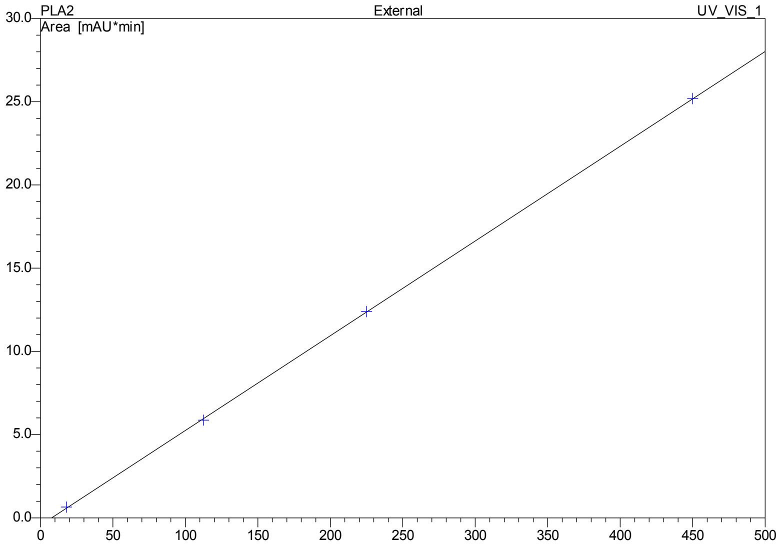

HPLC condition

HPLC: Thermo Dionex (Thermo Dionex, Sunnyvale, CA, USA)
Column: Agilent Zorbox 300SB-C18 (5 u, 4.6x150 mm)
Eluent:

min	Flow (ml/min)	A(0.3%TFA) %	Acetonitrile %
0	0.8	90	10
25	0.8	10	90
30	0.8	10	90
31	0.8	90	10
35	0.8	90	10

Detector: UV 220nm
Oven: 30 °C
